# Development of the Japanese 15D Instrument of Health-Related Quality of Life: Verification of Reliability and Validity among Elderly People

**DOI:** 10.1371/journal.pone.0061721

**Published:** 2013-04-16

**Authors:** Nozomi Okamoto, Akinori Hisashige, Yuu Tanaka, Norio Kurumatani

**Affiliations:** 1 Department of Community Health and Epidemiology, Nara Medical University, Kashihara, Nara, Japan; 2 Institute of Health Technology Assessment, Tokushima, Japan; 3 Department of Anesthesiology, Nara Medical University, Kashihara, Nara, Japan; Charité University Medicine Berlin, Germany

## Abstract

**Objective:**

The 15D is a self-administered questionnaire for assessment of health-related quality of life, which contains 15 questions with 5 response options each. This study was conducted to evaluate the reliability and validity of the Japanese 15D.

**Methods:**

The subjects were 430 community-dwelling elderly people. Each item of the 15D was scored on a 5-point Likert scale, with level 1 being the best, score 1. Reliability was assessed by determination of the internal consistency and test-retest reliability. Criterion-based validity was assessed using the Japanese version of the Nottingham Health Profile (NHP) and Tokyo Metropolitan Institute of Gerontology Index of Competence (TMIG index). Acceptability was assessed by inquiring about the time required to complete the questionnaire and the burden felt in responding to it.

**Results:**

The answers of 423 individuals who responded to all items were analyzed. The median time required to complete the questionnaire was 5.0 minutes, and the proportion of subjects who indicated that the questionnaire was easy to complete was 98.3%. The Cronbach’s alpha coefficients for all 15 items in the 2 surveys were 0.793 and 0.792, respectively. The intraclass correlation coefficients for the 15 items ranged from 0.44 to 0.72. In the relationship between the 15D and the NHP, the correlation coefficients between the corresponding domains were higher than those between non-corresponding domains. The prevalence of disability in higher-level functional capacity was higher in the “level 2 to 5” group than in the “level 1” group.

**Conclusions:**

The Japanese version of the 15D showed sufficient internal consistency and moderate repeatability. Because of the short time required to complete the Japanese 15D and the significant relationships between the scores on the 15D and the NHP, and between the 15D and higher-level functional capacity, the acceptability and validity of the Japanese 15D were considered to be sufficient.

## Introduction

In the last 3 decades, a number of questionnaires have been designed for quantitative assessment of the physical, mental and social aspects of health. Among the comprehensive health-related quality of life (HRQoL) questionnaires, the Medical Outcome Study 36-item short-form (SF-36) [1] and Nottingham Health Profile (NHP) [2]are representative profile index score measures, and EuroQol (EQ-5D) [3]is a representative single index score measure. The Japanese versions of these questionnaires, the originals of which are in English, have already been developed [4]–[6].

The original version of the 15D instrument used for assessment of the HRQoL, developed by Sintonen et al in Finland, is also in English [7]. It is a comprehensive, 15-dimensional, self-administered measure that can be used as both a profile and single index score measure for the adult population (aged over 15 years) [8]. The key dimensions include mobility, vision, hearing, breathing, sleeping, eating, speech, elimination, usual activities, mental function, discomfort and symptoms, depression, distress, vitality, and sexual activity. The response to each dimension is divided into 5 hierarchically structured levels. The best possible condition of an individual’s health is represented by level 1, the worst by level 5, and the rest describe intermediate health conditions between these 2 levels. The questionnaire is brief, and takes an average of 5 to 10 minutes to complete. The 15D has been utilized for surveys of general and patient populations [9]–[11] as a scale to compare different health conditions [12], [13], and also to measure the physical and mental effects of health-related interventions [14], [15].

The 15D may also be applicable in Japan as a rating scale for health status and as a predictor of health outcomes. We produced the Japanese version of the 15D after obtaining translation permission. The objective of this study was to evaluate the reliability and validity of the Japanese 15D. This process is needed before the development of a set of Japanese 15D preference weights, by which the responses to the dimensions are converted into a single-index score on a 0 to 1 scale (1 corresponds to full HRQoL, whereas 0 corresponds to death). More individuals with lower physical well-being or emotional well-being are included among elderly people or patients than among younger ages and healthy people. The subjects were drawn from the community-dwelling elderly.

## Methods

### Translation of the 15D into Japanese

The Japanese 15D Development Committee consisted of 3 Japanese men and 1 Japanese woman, who specialized in public health, epidemiology, and health economics. Two Japanese forward translations were independently undertaken by the experienced translators (H.A. and N.K.) among the committee members. At the committee meeting, the 2 translations were compared phrase-by phrase with the original English version, and a consensus on the best translation was arrived at. Back translations performed by 2 bilingual persons with English as their native language were confirmed a high level of agreement with the original version. The second consensus version was developed by slightly modifying the first consensus version.

We selected 8 respondents who were native speakers of Japanese (aged 44 to 75 years old, consisting of 4 males and 4 females, not involved in the health profession) for the respondent testing. The questionnaire was self-administered in the presence of a member of the committee (N.O.) and another collaborator. The respondents’ comments and queries about the second consensus version of the Japanese 15D were recorded. All 4 members of the committee then met to develop the third consensus version, taking into consideration the comments and queries of the respondents. We submitted reports of the forward and back translations and the respondent testing to the original author, Sintonen in Finland. The original author checked and approved the third consensus version. The reconciled version was then forwarded to a survey for determining its reliability and validity.

### Subjects

The subjects were 430 volunteer men and women aged 65 to 89 years old who belonged to the Senior Citizens Club Association in a local city of Nara prefecture, Japan. The participants were part of the cohort of the Fujiwara-kyo study, a larger prospective cohort study. Details of the Fujiwara-kyo study have been described elsewhere [16], [17].

This study was conducted with the approval of the Medical Ethics Committee of Nara Medical University. Written informed consent was obtained from each of the subjects prior to their participation in the study.

### Study Design

The Japanese version of the 15D was sent by mail to the subjects twice at a 4-week interval, and returned by mail (Survey 1 and Survey 2). To assess the acceptability of the Japanese 15D, the subjects were asked about the time that it took for them to complete the questionnaire and the burden with which they could answer the questions (extremely easy, very easy, relatively easy, relatively difficult, very difficult, or extremely difficult). Reliability was assessed by the internal consistency and test-retest methods. Criterion-based validity was assessed by examining the relationship of the 15D scores with the scores on the NHP and with the scores on the Tokyo Metropolitan Institute of Gerontology Index of Competence (TMIG index) [18]. The NHP and TMIG indexes and the self-reported disease history were included in the Survey 1 questionnaire.

The NHP contains 38 items grouped into 6 dimensions, namely, energy (3 items), pain (8 items), emotional reactions (9 items), sleep (5 items), social isolation (5 items), and physical mobility (8 items). All of the questions have a yes/no answer format (yes = 1, no = 0). To obtain the final score in each dimension, the sum in each dimension was multiplied by 100 and divided by the number of items in the dimension [19]. Possible scores ranged from 0 (denoting absence of distress) to 100 (denoting maximal distress).

The TMIG index is a scale developed in Japan consisting of 3 dimensions, namely, instrumental self-maintenance (5 items: using public transportation, shopping, preparing meals, paying bills, managing deposits), intellectual activity (4 items: filling out forms of pension, reading newspaper, reading books or magazines, being interested in stories or programs dealing with health), and social role (4 items: visiting the homes of friends, being called on for advise, visiting sick friends, initiating conversations with young people). The subjects were requested to choose the response option of “yes/can” (one point) or “no/cannot” (zero point) for each question. Lower scores indicated reduced higher-level functional capacity and a high mortality [20]. Disability was defined as a failure to achieve full scores in the respective dimensions [21].

### Statistical Analysis

Each item of the 15D was scored on a 5-point Likert scale (possible score range: 1 to 5, level 1 = score 1). Internal consistency was assessed by calculation of the Cronbach’s alpha coefficients, both overall and with the item deleted from the scale. Repeatability was assessed by the Bland-Altman method [22] and the 1-way random-effect model for single measures intraclass correlation coefficients (ICCs) [23].

Spearmen’s rank correlation coefficients between the 15D and NHP scores were calculated. Next, the subjects were divided into 2 groups, namely, “level 1” and “level 2 to 5” groups, for each answer of the 15D items. The difference in the prevalence of disability in higher-level functional capacity between the 2 groups was tested using the chi-square test.

Statistical analysis was performed using SPSS (version 17.0; SPSS Japan Inc., Tokyo, Japan). We calculated two-tailed P values in all of the analyses. The α level of significance was set at 0.05.

## Results

### Demographics of the Subjects

Of the 430 persons who agreed to cooperate in both surveys conducted 4-weeks apart, the answers of 423 (210 males and 213 females) who responded to all items were analyzed (98.4%). Table 1 shows the demographic characteristics of the subjects. The median age (25th percentile, 75th percentile) was 74.0 (71.0, 78.0) years. The proportions of subjects with a history of cancer, cerebrovascular disease, myocardial infarction, diabetes and hypertension were 11.6%, 5.0%, 1.9%, 9.5% and 37.8%, respectively.

**Table 1:** Demographic characteristics of the subjects (N = 423).

Variable		N	%
Gender	Male	210	49.6
	Female	213	50.4
Age (years)	65 to 69	54	12.8
	70 to 74	167	39.5
	75 to 79	140	33.1
	80 to 84	49	11.6
	85 to 89	13	3.1
Length of education	Less than 12 years	123	29.1
	12 years or more	300	70.9
Living status	Alone	69	16.3
	With family	354	83.7
Smoking habit	Never	37	8.7
	Ex-smoker	138	32.6
	Current smoker	248	58.6
Positive history of disease	Cancer	49	11.6
	Cerebrovascular disease	21	5.0
	Myocardial infarction	8	1.9
	Diabetes mellitus	40	9.5
	Hypertension	160	37.8

### Assessment for Acceptability

The median value (25th percentile, 75th percentile) of the time required to complete the 15D was 5.0 (3.0, 7.0) minutes. As to the subjective burden felt in answering the 15D, the highest proportion (49.6%) of subjects selected “extremely easy”, followed by “very easy” (32.9%), “relatively easy” (15.8%), and “relatively difficult” (1.7%). Both “very difficult” and “extremely difficult” were selected by 0.0% of the subjects.

### Descriptive Statistics and Internal Consistency

Table 2 summarizes results of the 15D-dimension descriptive statistics and internal consistency. Both in Survey 1 and Survey 2, the proportion of subjects who selected level 1 or level 2 exceeded 90%, excluding the sexual activity dimension. In addition, more than 80% of the subjects selected level 1 in hearing, eating, speech, elimination, and usual activities, showing a ceiling effect. The Cronbach’s alpha coefficients for all 15 items were 0.793 and 0.792 in Survey 1 and Survey 2, respectively. The alpha coefficients were reduced when 1 dimension was deleted from the scale. Only the exclusion of the eating and sexual activity dimensions resulted in an improved Cronbach’s alpha, implying that their presence decreased overall reliability.

**Table 2:** Descriptive statistics and internal consistency of items on the 15D.

	Response frequency (%)										Cronbach’s α coefficients	
	Survey 1					Survey 2					(item deleted)	
	1	2	3	4	5	1	2	3	4	5	Survey 1	Survey 2
Mobility	74.5	22.9	2.4	0.2	0	79.4	19.4	0.9	0	0.2	0.780	0.777
Vision	69.5	26.2	2.6	1.7	0	79.2	19.1	1.7	0	0	0.791	0.787
Hearing	81.6	14.7	3.8	0	0	81.3	14.9	3.5	0.2	0	0.791	0.790
Breathing	70.0	26.7	3.1	0.2	0	70.4	27.7	1.2	0.5	0.2	0.779	0.773
Sleeping	54.1	36.6	3.3	5.7	0.2	61.2	30.7	2.8	5.2	0	0.788	0.792
Eating	99.5	0.5	0	0	0	99.5	0.5	0	0	0	0.796	0.795
Speech	93.1	5.4	1.4	0	0	93.1	5.7	0.9	0.2	0	0.784	0.781
Elimination	81.6	14.7	3.5	0	0.2	81.3	15.1	3.5	0	0	0.781	0.777
Usual activities	88.7	10.4	0.7	0.2	0	89.8	9.2	0.5	0.5	0	0.778	0.775
Mental function	73.3	26.5	0	0.2	0	75.2	24.6	0	0	0.2	0.783	0.784
Discomfort and symptoms	50.1	46.8	2.6	0	0.5	56.7	39.2	3.8	0	0.2	0.774	0.775
Depression	71.6	26.5	1.7	0	0.2	73.8	24.3	1.7	0.2	0	0.771	0.773
Distress	54.4	42.3	2.8	0.5	0	54.6	42.3	2.6	0.5	0	0.770	0.769
Vitality	51.3	43.5	4.3	0.7	0.2	51.1	44.4	4.3	0.2	0	0.757	0.765
Sexual activity	49.9	27.9	9.0	5.9	7.3	48.2	28.6	9.0	8.0	6.1	0.792	0.800

### Repeatability

Table 3 shows item-scale repeatability analyses. The lower limit of agreement and the upper limit of agreement were approximately −1 and +1, respectively, in the 14 items excluding the sexual activity dimension. Those in sexual activity dimension were approximately −2 and +2, respectively. The range of ICCs for the 15 items was between 0.44 and 0.72, and moderate repeatability was observed in all dimensions.

**Table 3:** Repeatability of items on the 15D.

	Bland-Altman analysis			Intraclass correlation
	The lower limit of agreement	The upper limit of agreement		coefficients
	(95% confidence interval)		(95% confidence interval)	(95% confidence interval)
Mobility	−0.79(−0.87 to −0.72)		0.92(0.84 to 0.99)	0.60(0.54 to 0.66)
Vision	−0.93(−1.03 to −0.84)		1.21(1.12 to 1.30)	0.47(0.39 to 0.54)
Hearing	−0.80(−0.87 to −0.74)		0.79(0.73 to 0.86)	0.68(0.62 to 0.72)
Breathing	−1.06(−1.15 to −0.97)		1.09(1.00 to 1.18)	0.50(0.42 to 0.57)
Sleeping	−1.07(−1.17 to −0.98)		1.26(1.16 to 1.36)	0.72(0.67 to 0.76)
Eating	−0.13(−0.15 to −0.12)		0.13(0.12 to 0.15)	0.50(0.42 to 0.57)
Speech	−0.79(−0.85 to −0.73)		0.69(0.63 to 0.75)	0.62(0.56 to 0.68)
Elimination	−0.89(−0.97 to −0.81)		0.90(0.82 to 0.98)	0.60(0.54 to 0.66)
Usual activities	−0.77(−0.83 to −0.70)		0.78(0.72 to 0.85)	0.44(0.36 to 0.51)
Mental function	−0.83(−0.90 to −0.76)		0.86(0.79 to 0.94)	0.57(0.50 to 0.63)
Discomfort and symptoms	−1.04(−1.13 to −0.94)		1.16(1.07 to 1.25)	0.56(0.49 to 0.62)
Depression	−0.89(−0.97 to −0.81)		0.94(0.86 to 1.01)	0.59(0.52 to 0.65)
Distress	−1.02(−1.11 to −0.94)		1.03(0.95 to 1.12)	0.59(0.52 to 0.65)
Vitality	−1.12(−1.22 to −1.03)		1.15(1.05 to 1.25)	0.55(0.48 to 0.62)
Sexual activity	−1.90(−2.06 to −1.74)		1.85(1.69 to 2.01)	0.69(0.64 to 0.74)

### Criterion-based Validity

Table 4 shows the correlation between the 15D and NHP scores. Significant correlations were observed between mobility in the 15D and physical mobility in the NHP (Spearman’s rank correlation coefficient: 0.62), between sleeping and sleep (0.58), between usual activities and energy (0.40), between mental function and social isolation (0.42), between discomfort-and-symptoms and pain (0.47), between depression and emotional reactions (0.49), between distress and emotional reactions (0.49), and between vitality and energy (0.50). The correlation coefficients between these corresponding domains were higher than those between non-corresponding domains.

**Table 4:** Spearman’s rank correlation coefficients between the 15D and Nottingham Health Profile dimensions.

	Nottingham Health Profile					
15D	Energy	Pain	Emotional reactions	Sleep	Social isolation	Physical mobility
Mobility	0.32^b^	0.41^b^	0.23^b^	0.18^b^	0.21^b^	***0.62*** b
Vision	0.14^b^	0.22^b^	0.09	0.09	0.02	0.18^b^
Hearing	0.14^b^	0.07	0.04	0.16^b^	0.15^b^	0.22^b^
Breathing	0.27^b^	0.22^b^	0.18^b^	0.20^b^	0.23^b^	0.31^b^
Sleeping	0.25^b^	0.22^b^	0.31^b^	***0.58*** b	0.15_b_	0.17^b^
Eating	0.12^b^	0.04	0.05	0.03	0.09	0.12^a^
Speech	0.27^b^	0.17^b^	0.22^b^	0.13^b^	0.33^b^	0.26^b^
Elimination	0.25^b^	0.16^b^	0.32^b^	0.24^b^	0.24^b^	0.22^b^
Usual activities	***0.40*** b	0.29^b^	0.30^b^	0.15^b^	0.33^b^	0.35^b^
Mental function	0.26^b^	0.21^b^	0.27^b^	0.16^b^	***0.42*** b	0.26^b^
Discomfort and symptoms	0.37^b^	***0.47*** b	0.17^b^	0.21^b^	0.26^b^	0.35^b^
Depression	0.33^b^	0.26^b^	***0.49*** b	0.34^b^	0.41^b^	0.29^b^
Distress	0.32^b^	0.28^b^	***0.49*** b	0.35^b^	0.33^b^	0.27^b^
Vitality	***0.50*** b	0.36^b^	0.35^b^	0.30^b^	0.42^b^	0.45^b^
Sexual activity	0.34^b^	0.24^b^	0.28^b^	0.26^b^	0.29^b^	0.32^b^

a P<0.05,

b P<0.01.

In Table 5, higher-level functional capacity is compared between the “level 1” and “level 2 to 5” groups in the 15D. Level 1 and level 2 were separated, because the responses were distributed mainly in level 1 and 2. The prevalence of disability in instrumental self-maintenance was significantly higher in the “level 2 to 5” group than in the “level 1” group for 8 dimensions of the 15D, the prevalence of disability in intellectual activity for 6 dimensions, and the prevalence of disability in social role for 9 dimensions.

**Table 5:** Comparison of higher-level functional capacity between “level 1” and “level 2 to 5” in the 15D.

			Disability (presence, %)					
			Instrumental self-maintenance		Intellectual activity		Social role	
15D		N	%	P value^a^	%	P value^a^	%	P value^a^
Mobility	Level 1	336	4.8	<0.01	18.7	0.18	19.0	0.14
	Level 2–5	87	16.5		25.6		26.7	
Vision	Level 1	335	5.1	<0.01	15.9	<0.01	18.6	0.05
	Level 2–5	88	14.8		36.4		28.4	
Hearing	Level 1	344	5.0	<0.01	18.6	0.12	18.3	0.02
	Level 2–5	79	16.7		26.9		30.8	
Breathing	Level 1	298	6.4	0.41	16.8	0.01	18.1	0.06
	Level 2–5	125	8.9		28.2		26.6	
Sleeping	Level 1	259	6.9	0.85	21.6	0.38	18.1	0.14
	Level 2–5	164	7.4		17.8		24.5	
Eating	Level 1	421	7.1	1.00	20.2	1.00	20.5	0.37
	Level 2–5	2	0		0		50.0	
Speech	Level 1	394	5.3	<0.01	19.0	0.05	18.0	<0.01
	Level 2–5	29	33.3		35.7		57.1	
Elimination	Level 1	344	5.2	<0.01	20.1	1.00	16.9	<0.01
	Level 2–5	79	15.4		20.5		37.2	
Usual activities	Level 1	380	5.8	<0.01	18.7	0.04	18.4	<0.01
	Level 2–5	43	19.5		33.3		40.5	
Mental function	Level 1	318	4.4	<0.01	17.6	0.03	15.7	<0.01
	Level 2–5	105	15.4		27.9		35.6	
Discomfort and symptoms	Level 1	240	6.7	0.71	19.2	0.62	17.5	0.09
	Level 2–5	183	7.7		21.4		24.7	
Depression	Level 1	312	6.7	0.67	17.3	0.02	15.4	<0.01
	Level 2–5	111	8.3		28.2		35.5	
Distress	Level 1	231	6.5	0.70	16.5	0.04	15.2	<0.01
	Level 2–5	192	7.9		24.6		27.2	
Vitality	Level 1	216	5.6	0.26	19.0	0.55	14.8	<0.01
	Level 2–5	207	8.8		21.4		26.7	
Sexual activity	Level 1	204	3.9	0.01	17.2	0.15	12.7	<0.01
	Level 2–5	219	10.1		22.9		28.0	

a P values were calculated by the chi-square test.

## Discussion

The results of the present study show that the development of the Japanese 15D has been successful. We found the Japanese version of 15D easy to administer and acceptable for community-dwelling elderly people.

The Turkish and Greek versions of 15D have been developed [24], [25] in hospital-based surveys of patients with type 2 diabetes and coronary artery disease, respectively. The Cronbach’s alpha coefficients for all items were 0.99 and 0.84 in the Turkish and Greek versions, respectively. The alpha values for all items were 0.793 and 0.792 in the 2 surveys of this study (Table 2). These values were lower than those reported from previous studies. However, sufficient internal consistency was confirmed because the 0.70 criterion was exceeded. Eating and sexual activity dimensions decreased the overall internal consistency. The decreases were small, and there was no problem in including the 2 items in the Japanese version of 15D.

In a report of the Greek version of the 15D [25], the proportion of subjects who selected level 3 to level 5 (poor health conditions) was approximately 10 to 60% for each of the 15 items. The full range of possible responses was used. On the other hand, in the present study, almost no subject selected level 3 to level 5. The proportion of subjects who selected level 1 or level 2 exceeded 90% for 14 items, excluding the sexual activity dimension; thus, the responses were unevenly distributed (Table 2). Individual differences were small. We speculate that this is because the subjects in this study were volunteers and community-dwelling independent people in relatively good health. The errors of repeated measurements showed by the limit of agreement (Table 3) would be relatively large compared to individual differences. This would result in the lower ICC values. The repeatability was not considered to be poor, although the ICC values were not more than 0.72 (Table 3).

The 15D shows greater discriminatory power in terms of the “ceiling” and “floor” effects than the EQ-5D and NHP [26]–[28]. In the results of this study, a ceiling effect was observed in 5 items, namely, hearing, eating, speech, elimination, and usual activities dimensions (Table 2). A ceiling effect has been also observed for the items of SF-36, namely, “walking one hundred meters” and “bathing or dressing” [29]. It is considered that items related to the basic activities of daily living tend to show a ceiling effect in community-based surveys.

In the relationship between the Japanese 15D and the NHP, the correlation coefficients between the corresponding domains were higher than those between non-corresponding domains (Table 4). The prevalence of disability in higher-level functional capacity was higher in the “level 2 to 5” group than in the “level 1” group (Table 5). The criterion-based validity was confirmed between the 15D and the NHP, and between the 15D and the TMIG-index.

Three limitations of the present study merit consideration. First, longitudinal construct validity and responsiveness were not addressed. It is necessary to investigate whether the instrument can detect changes in the health status over time. Second, the subjects in the present study were limited to the community-dwelling elderly aged 65 years or older. This limits the conclusions that can be drawn about the validity of the measure for use in other groups. Third, distribution of item selection was skewed. It is necessary to verify the validity in subjects over a wider age range, including middle-aged and young people, but, if so, distribution of item selection will be more skewed toward healthy responses. It may be proper that we conducted the survey on elderly people.

### Conclusions

Within the limitations of the study design mentioned above, the results of this study indicate that the Japanese 15D has sufficient internal consistency and moderate repeatability. Because of the short time required to complete the Japanese 15D, the significant correlation between the corresponding domains of the 15D and the NHP, and the significant relationship between the 15D and higher-level functional capacity, the levels of acceptability and validity of the Japanese 15D for the community-dwelling elderly are considered sufficient. Further studies for the development of the scoring algorithm and a single-index score for the Japanese version of the 15D instrument are needed.

## Supporting Information

Appendix S1 The Japanese version of the 15D.
(PDF)
