# Supplementary material for: Development of the Japanese 15D Instrument of Health-Related Quality of Life: Verification of Reliability and Validity among Elderly People
Source: PLoS One. 2013 Apr 16;8(4):e61721. doi: 10.1371/journal.pone.0061721 (PMC3627920; doi:10.1371/journal.pone.0061721)
Supplement: Appendix S1 — The Japanese version of the 15D. (PDF) [file pone.0061721.s001.pdf]

Appendix S1

生活の質についての質問表（15D）

それぞれの質問について、すべての選択肢を読んで、**あなたの現在の健康状態**を最もよくあらわしている選択肢に、〇をつけて下さい。15 の質問すべてについて、同じようにつづけて下さい。それぞれの質問について**一つ**だけ答えてください。

質問1．移動

ア．　屋内や屋外、階段を、普通に（苦もなく）歩くことができる。

イ．　屋内は苦もなく歩くことができる。しかし、屋外や階段では少し難しい。

ウ．　屋内は助けなしに(補助具を使っても、使わなくても)歩くことができる。
しかし、屋外や階段ではかなり難しかったり、人の助けを必要としたりする。

エ．　屋内は、人の助けなしには歩くことができない。

オ．　完全に寝たきりであり、動きまわることができない。

質問2．視力

ア．　普通に見ることができる。つまり、苦もなく（メガネをかけても、かけなくても）新聞やテレビの字幕を読むことができる。

イ．　新聞やテレビの字幕を読むのが、少し難しい（メガネをかけても、かけなくても）。

ウ．　新聞やテレビの字幕を読むのが、かなり難しい（メガネをかけても、かけなくても）。

エ．　新聞やテレビの字幕を読むことはできない（メガネをかけても、かけなくても）。しかし、案内なしに歩き回ることができる。

オ．　十分には見えないので、案内人なしには歩き回れない。つまり、ほとんどあるいは完全に失明している。

質問3．聴力

ア．　普通に、つまり普通の話し声を聞くことができる(補聴器を使っても、使わなくても)。

イ．　普通の話し声を聞くのが、少し難しい。

ウ．　普通の話し声を聞くのが、かなり難しい。会話では、普通よりも大きな声で話してもらう必要がある。

エ．　大きな声でもあまり聞こえない。ほとんど耳が聞こえない。

オ．　全く耳が聞こえない。

質問4. 呼吸

ア. 普通に息をすることができる。つまり、息切れしたり、息をするのが苦しくなったりすることはない。

イ. きつい仕事やスポーツをするとか、平地やわずかな登り坂を急ぎ足に歩くと、息が切れる。

ウ. 平地を同じような年齢の人と同じ早さで歩くと、息が切れる。

エ. 軽い活動の後、たとえば、体を洗ったり服を着たりした後でも、息が切れる。

オ. ほとんどいつも、じっとしているときでも、息をするのが苦しい。

質問5. 睡眠

ア. 普通に眠ることができる。つまり、睡眠に問題は無い。

イ. 睡眠に少し問題がある。たとえば、寝つきにくかったり、ときどき夜中に目が覚めたりする。

ウ. 睡眠にかなり問題がある。たとえば、眠りが妨げられたり、じゅうぶん眠れなかったと感じたりする。

エ. 睡眠に大きな問題がある。たとえば、睡眠薬をたびたび、あるいは日常的にのまなくてはならない。そうしないと、いつも夜中やとても朝早くに目が覚める。

オ. ひどい不眠に苦しんでいる。たとえば、睡眠薬を限度までのんでもほとんど眠れなかったり、ほとんど一晩中、目が覚めていたりする。

質問6. 食事

ア. 普通に食べることができる。つまり、人の助けなしに。

イ. 自分で食べるのが、すこし難しい（たとえば、のろのろと、ぎこちなく、ふるえて。あるいは、特別な食器を使って）。

ウ. 食べるのに、いくらか人の助けを必要とする。

エ. 自分でまったく食べることができない。そのため、人に食べさせてもらわなければならない。

オ. まったく食べることができない。そのため、点滴や管から栄養を受けている。

質問7. 話し

ア. 普通に話すことができる。つまり、はっきりと、聞こえるように、すらすらと。

イ. 話すのが少し難しい。たとえば、ときおり言葉を捜したり、ぶつぶつ言ったり、高低が変わる。

ウ. 自分の考えを人に分からせることができる。しかし、自分の話は、とりとめがなく、口ごもり、つまったり、どもったりする。

エ. 自分の話は、ほとんどの人にとって理解するのがたいへん難しい。

オ. 身ぶりでしか、自分の考えを人に分からせることができない。

質問8. 排泄

ア.　普通に、問題なく、排尿と排便ができる。

イ.　排尿や排便に少し問題がある。たとえば、尿が出にくかったり、ゆるい便やかたい便であったりする。

ウ.　排尿や排便にかなり問題がある。たとえば、時々「もらし」たり、ひどい便秘や下痢をしたりする。

エ.　排尿や排便に重大な問題がある。たとえば、頻繁に「もらし」たり、導尿や浣腸を必要としたりする。

オ.　排尿や排便が、まったく自分の思うようにならない。

質問9. 日常活動

ア.　日常活動（たとえば、仕事、勉学、家事、余暇活動）を苦もなくすることができる。

イ.　日常活動が、あまり上手くできなかったり、すこし難しかったりする。

ウ.　日常活動が、ほとんど上手くできなかったり、かなり難しかったり、完全でなかったりする。

エ.　以前できていた日常活動の一部分しか、やりとげることができない。

オ.　以前できていた日常活動は、何もやりとげることができない。

質問 10. 精神機能

ア.　はっきりと、すじ道を立てて考えることができる。記憶の働きは良い。

イ.　はっきりと、すじ道を立てて考えるのが、すこし難しかったり、記憶がときどきまちがったりする。

ウ.　はっきりと、すじ道を立てて考えるのが、かなり難しかったり、記憶が多少そこなわれていたりする。

エ.　はっきりと、すじ道を立てて考えるのが、たいへん難しかったり、記憶がひどくそこなわれていたりする。

オ.　ずっと頭が混乱したままであり、場所と時間の見当を失っている。

質問 11. 不快と症状

ア.　からだに、不快や症状はまったく無い。たとえば、痛み、うずき、はきけ、かゆみなど。

イ.　からだに、かるい不快や症状がある。たとえば、痛み、うずき、はきけ、かゆみなど。

ウ.　からだに、かなり不快や症状がある。たとえば、痛み、うずき、はきけ、かゆみなど。

エ.　からだに、ひどい不快や症状がある。たとえば、痛み、うずき、はきけ、かゆみなど。

オ.　からだに、耐え難い不快や症状がある。たとえば、痛み、うずき、はきけ、かゆみなど。

質問 12. うつ

ア.　悲しみや、ゆううつ、落ち込みをまったく感じない。

イ.　悲しみや、ゆううつ、落ち込みをすこし感じている。

ウ.　悲しみや、ゆううつ、落ち込みをかなり感じている。

エ.　悲しみや、ゆううつ、落ち込みをたいへん感じている。

オ.　悲しみや、ゆううつ、落ち込みをきわめてつよく感じている。

質問 13. なやみ

ア.　ストレスや、いらいら、不安をまったく感じない。

イ.　ストレスや、いらいら、不安をすこし感じている。

ウ.　ストレスや、いらいら、不安をかなり感じている。

エ.　ストレスや、いらいら、不安をたいへん感じている。

オ.　ストレスや、いらいら、不安をきわめてつよく感じている。

質問 14. 活力

ア.　健康で活力があると感じている。

イ.　疲れや、飽き、弱りをすこし感じている。

ウ.　疲れや、飽き、弱りをかなり感じている。

エ.　疲れや、飽き、弱りをたいへん感じており、ほとんど疲れはてている。

オ.　疲れや、飽き、弱りをきわめてつよく感じており、まったく疲れはてている。

質問 15. 性活動

ア.　健康状態は、性活動に不都合な影響をまったく及ぼしていない。

イ.　健康状態は、性活動にすこし影響を及ぼしている。

ウ.　健康状態は、性活動にかなり影響を及ぼしている。

エ.　健康状態のため、性活動がほとんどできない。

オ.　健康状態のため、性活動ができない。
